# Community Outbreak of OXA-48–Producing *Escherichia coli* Linked to Food Premises, New Zealand, 2018–2022

**DOI:** 10.3201/eid3107.250289

**Published:** 2025-07

**Authors:** Craig N. Thornley, Matthew Kelly, Max Bloomfield, Loushy Mangalasseril, Annette Nesdale, Claire Underwood, Kristin Dyet, Juliet Elvy, Jenny Szeto, Hermes Perez, Xioayun Ren, Rosemary Woodhouse, Rhys T. White

**Affiliations:** Health New Zealand Te Whatu Ora, Lower Hutt, New Zealand (C.N. Thornley, M. Kelly, L. Mangalasseril, A. Nesdale, C. Underwood); Awanui Labs Wellington, Wellington, New Zealand (M. Bloomfield); Institute of Environmental Science and Research, Porirua, New Zealand (K. Dyet, J. Elvy, J. Szeto, H. Perez, X. Ren, R. Woodhouse, R.T. White); Awanui Labs Dunedin, Dunedin, New Zealand (J. Elvy)

**Keywords:** bacteria, food safety, antimicrobial resistance, carbapenem-resistant Enterobacteriaceae, Escherichia coli, disease outbreaks, infectious disease transmission, foodborne diseases, molecular epidemiology, New Zealand

## Abstract

In New Zealand, OXA-48–producing *Escherichia coli* is uncommon and typically associated with international travel. We investigated a cluster of 25 patients without recent travel history from Hutt Valley health district, New Zealand, who had multilocus sequence type 131 OXA-48–producing *E. coli* during August 2018–December 2022. Eighteen had been admitted to Hutt Valley Hospital but did not share a common ward or hospital service. Eighteen had visited the same community-based commercial food premises (premises A); 7 of those had not been admitted to Hutt Valley Hospital. An inspection of premises A revealed multiple hazards, primarily around staff hand hygiene. Four food handlers were colonized with OXA-48–producing *E. coli*; whole-genome sequencing confirmed genomic links between case and food handler strains, with possible introduction to New Zealand circa 2017. Community-based food premises have a role in propagating OXA-48–producing *E. coli* in high-income countries, requiring consideration in control strategies.

Carbapenemase-producing Enterobacterales (CPE) hydrolyze and inactivate carbapenems, the β-lactam antibiotic drugs with the broadest coverage spectrum, limiting treatment options for serious gram-negative infections and increasing rates of illness and death (*1*,*2*). The OXA-48 carbapenemase and variants (collectively termed OXA-48–like enzymes) demonstrate low-level hydrolysis of carbapenems. Despite that, they represent a potential source of clinical failure for β-lactams (*3*). They are also capable of spreading between strains and species because they are typically found on mobile genetic elements (*4*). Increasing global prominence of OXA-48–like carbapenemases has been attributed to horizontal spread through plasmids and vertical spread with multidrug-resistant clones (*5*), and *bla*_OXA-48_ is one of the carbapenemase genes increasingly detected in *Escherichia coli* sequence type (ST) 131, a high-risk extraintestinal pathogenic *E. coli* lineage (*6*). In New Zealand (Aotearoa), CPE identification has increased since it was first detected in 2009 (*7*), but it remains uncommon and is typically associated with international travel or contact with travelers (*7*,*8*).

Transmission of CPE in healthcare settings has been well described (*9*,*10*), and guidance on CPE control has consequently focused on those environments (*11*). Although recognition of the importance of community acquisition of CPE is emerging (*12*,*13*), studies of transmission pathways have largely focused on within-household contact (*14*,*15*). CPE transmission might be more dynamic than current evidence suggests; research on other antibiotic-resistant Enterobacterales points to multifaceted source attribution (*16*,*17*), and similar patterns might exist for CPE. CPE have been detected in food-producing animals (*18*), in food (*19*), and among food handlers (*20*), and hospital foodborne transmission has occurred (*21*).

In August 2018, a series of patients living in Hutt Valley health district, New Zealand, without recent international travel history were found to have clinical infection with or carriage of OXA-48–producing *E. coli*, all of which were found to be multilocus ST 131. Because some of the patients had no recent hospitalizations, an investigation was undertaken to identify and control a possible common source.

## Methods

### Setting

Hutt Valley health district consists of Lower Hutt and Upper Hutt local government areas and has a population of ≈150,970, which is predominantly urban and suburban. The district is served by a single main 322-bed public hospital, Hutt Valley Hospital (HVH); hospital inpatient wards consist predominantly of 4-bed rooms with a shared bathroom. Community and hospital diagnostic laboratory services for the district are provided by a single laboratory, Awanui Labs Wellington (an International Accreditation New Zealand ISO15189 accredited medical laboratory). Vitek MS is used for organism identification and Vitek II for antimicrobial susceptibility testing (both bioMérieux, https://www.biomerieux.com), using the AST N311 card and following European Committee on Antimicrobial Susceptibility Testing guidelines. All clinically significant Enterobacterales grown from clinical samples are screened for carbapenemase production according to guidelines (*22*) (Appendix).

### Case Detection and Investigation

We defined cases as OXA-48–producing *E. coli* ST131 obtained from specimens collected for diagnostic purposes, routine surveillance of HVH inpatients, or hospital contact screening in Hutt Valley health district residents who had no recent international travel (Appendix). We compiled a case dataset to investigate CPE acquisition risk factors. We obtained data from hospital records for all inpatient admission episodes (defined as a hospital stay of >4 hours) during January 2015–February 2023. Data included admission dates, clinical services, and hospital locations, which we analyzed to determine whether >2 cases had concurrent admissions in the same ward. We interviewed case-patients using a schedule of questions on visits to ready-to-eat food premises, travel, use of health services abroad, household contacts, and other factors for a period covering the preceding 4 years (Appendix Table 1).

### Enhanced Community Surveillance

To assess spread of the organism in the wider community, we conducted enhanced surveillance for CPE in routinely submitted urine and stool specimens for a fixed-term 8-month period during 2020–2021. This program applied lower laboratory thresholds for CPE screening than were used in routine processing of those samples (Appendix). 

### Environmental Investigation

We identified a community-based commercial food premises serving ready-to-eat food (premises A) from case interviews as a potential common exposure source. Food Act Officers undertook an environmental inspection in December 2018, with support from public health officials, that focused on hand hygiene, food storage, food preparation practices, customer bathrooms, and use of imported food. In July 2019, kitchen and bathroom surfaces, frequently touched items, and food samples were tested for *E. coli* (regardless of resistance phenotype) and CPE; testing of surfaces was repeated in January 2021 with the addition of water samples from kitchen sink drains, and further testing of surfaces occurred in June 2021 (Appendix). In May 2019 and November 2020, food handlers were invited to provide stool specimens to test for CPE; samples were processed using the same method as for the enhanced community surveillance, using an extended-spectrum β-lactamase (ESBL)/vancomycin-resistant *Enterococcus *(VRE) chromogenic agar (CHROMagar, https://www.chromagar.com) (Appendix). We interviewed those who tested positive regarding their travel history and healthcare use and collected data on their hospital attendance.

### Confirmatory Testing and Whole-Genome Sequencing

We submitted all suspected CPE for confirmation and whole-genome sequencing (WGS) using the NextSeq 550 system (Illumina, https://www.illumina.com), generating 2 × 151-bp paired-end reads, at the New Zealand public health laboratory, the Institute of Environmental Science and Research, in Porirua, New Zealand. We also performed long-read sequencing (Oxford Nanopore Technologies, https://nanoporetech.com) on the index isolate to allow genome assembly and resolution of plasmid structure. We undertook nanopore read quality control, Illumina sequencing, genome assembly, multilocus sequence typing, virulence and antibiotic resistance gene genotyping, public data curation, and attempted estimation of the cluster emergence date (Appendix). 

### Ethics

The investigation was defined by the NZ Health and Disability Ethics Committees as a public health investigation and therefore approval was not required. CPE carriage is not a notifiable disease in New Zealand, and informed consent was obtained from all patients to access medical records and conduct case interviews.

## Results

### Case Investigation

**Figure 1:**
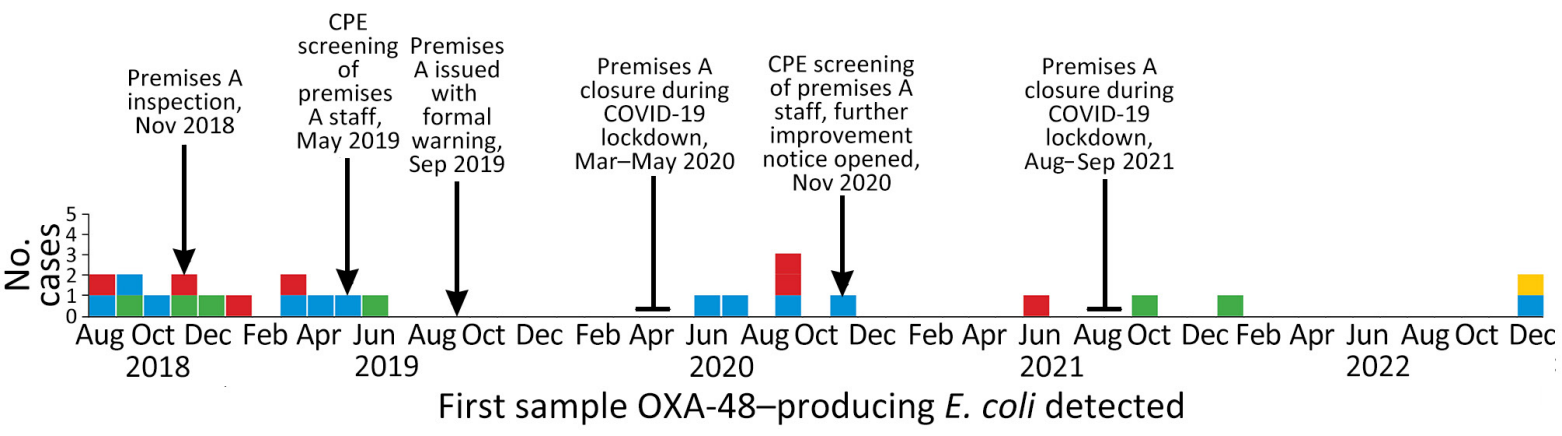
Epidemic curve of 25 cases of OXA-48–producing *Escherichia coli*, by month of sample collection, in study of community outbreak linked to food premises, Hutt Valley, New Zealand, August 2018–December 2022. Cases are categorized according to history of exposure to a community-based food premises (premises A) and history of inpatient admission to Hutt Valley Hospital (HVH) in the 4-year period before detection: 7 had been exposed to premises A but not HVH (red), 11 had been exposed both to premises A and to HVH (blue), 6 had been exposed to HVH but not to premises A (green), and 1 person had been exposed to HVH but premises A exposure was unknown (yellow). CPE, carbapenemase-producing Enterobacterales.

During August 2018–December 2022, we identified 25 cases (Figure 1). All were detected through use of routine testing or surveillance protocols (Appendix). Of the 25 cases, 18 were first detected from urine samples; 11 were associated with uncomplicated urinary tract infections, 6 with asymptomatic bacteriuria, and 1 with pyelonephritis secondary to existing renal disease. Of the remaining 7 cases, 4 were detected on reflex testing of loose stool samples, 1 through a hospital contact screening stool sample, 1 through a blood culture associated with urosepsis, and 1 from tissue biopsy. Seven of the total cases were identified from community-collected samples (all urine); the remaining 18 cases were detected from samples collected in the hospital (11 from urine samples and 7 from nonurine samples). The median age of case-patients was 74 (range 37–94) years (Table 1). No single ward or hospital location was common to the 18 case-patients admitted to HVH. In 4 instances, 2 cases had concurrent same-ward admissions; in 3 instances, case-patients were admitted to a ward from which another case-patient had been discharged up to 7 days previously; those instances occurred in 7 different wards.

**Table 1:** Characteristics of 25 case-patients with OXA-48–producing *Escherichia coli* in study of community outbreak linked to a food premises, Hutt Valley, New Zealand, August 2018–December 2022

Category	No. (%) patients
Sex	
F	18 (72.0)
M	7 (28.0)
Ethnicity	
New Zealand European	20 (80.0)
Māori	4 (16.0)
Samoan	1 (4.0)
Specimen positive for OXA-48–producing *E. coli*	
Urine sample collected in community	7 (28.0)
Urine sample collected in hospital	11 (44.0)
Stool sample collected in hospital through loose stool	4 (16.0)
Blood culture	1 (4.0)
Tissue specimen	1 (4.0)
Stool sample collected in hospital through contact tracing	1 (4.0)
Infection associated with OXA-48–producing *E. coli*	
Uncomplicated urinary tract infection	11 (44.0)
Pyelonephritis/urosepsis	2 (8.0)
Osteomyelitis	1 (4.0)
No illness due to OXA-48–producing *E. coli*	11 (44.0)
Hospital exposure	
Hutt Valley Hospital admission	18 (72.0)
Wellington Regional Hospital admission	4 (16.0)
Any hospital admission in Wellington region	21 (84.0)
Healthcare use outside region or outside New Zealand	0
Other selected exposures*	
Exposure to specific community food premises (premises A)	18 (75.0)
Untreated drinking water	1 (4.2)
Recreational water exposure	5 (20.8)
Exposure to domestic animals	8 (33.3)
Exposure to farm animals	2 (8.3)

*Data on nonhealthcare exposures were available for only 24 cases, because 1 case-patient died before an interview could be undertaken.

We identified a total of 44 ready-to-eat food premises in the exposure histories of the 24 case-patients interviewed (1 person died before interview). Only 2 premises had been visited by >3 case-patients: of those, 1 had been visited by 18 case-patients (premises A); the other had been visited by 7 case-patients, all of whom had also visited premises A. Of the 18 persons who had visited premises A, 7 had no HVH admission history. The time interval between their most recent premises A visit and collection of the clinical specimen that tested positive for OXA-48–producing *E. coli* ranged from <1 month to >48 months; 50% had visited within the previous 2-month period (Appendix Table 2).

Of the 7 case-patients without HVH admission, 4 had received no hospital-level healthcare since 2015. The remaining 3 case-patients had either been inpatients (n = 2) or outpatients (n = 1) at other hospitals in the region. Of the 7 case-patients with HVH admission but no definite premises A exposure, 2 had concurrent same-ward hospitalization with case-patients who had previous exposure to premises A. Of the 11 case-patients with both hospital admission and premises A exposure (Table 2), 2 had had concurrent admission with other case-patients before those persons’ CPE diagnosis, none of whom had previous premises A exposure.

**Table 2:** Categorization of 24 case-patients with OXA-48–producing *Escherichia coli*, according to history of exposure to a community-based food premises and history of HVH admission, in study of community outbreak linked to a food premises, Hutt Valley, New Zealand, August 2018–December 2022*

Year of collection of sample from which OXA-48–producing *E. coli* was detected	No. with premises A exposure but no HVH inpatient admission	No. with premises A exposure and HVH inpatient admission	No. with HVH inpatient admission but no premises A exposure	Total no.
2018	2	3	3	8
2019	2	3	1	6
2020	2	4	–	6
2021	1	–	1	2
2022	–	1	1	2
Total	7	11	6	24

*Categorization of premises A and HVH exposure was only possible for 24 cases, because 1 case-patient died before interview could be undertaken. HVH, Hutt Valley Hospital; premises A, community-based food premises.

### Enhanced Community Surveillance

Over the 8-month enhanced community surveillance program, we screened 217 stool samples and 2,050 urine samples from patients residing in the target suburbs. We detected no OXA-48–producing organisms in those samples.

### Environmental Investigation

Premises A was registered with the local government and commenced operation in 2017 providing ready-to-eat food. Gender-specific toilets on the premises were used both by staff and customers. Multiple food safety concerns were identified during the first inspection in November 2018. The kitchen handwashing sink was not being used because of negligible water pressure and obstructed access. Food handlers used gloves, but glove changes and performance of hand hygiene measures were infrequent. Chopping boards were used without apparent segregation in usage between uncooked or cooked food, and equipment and food supplies were not well organized. Personal clothing items were present in the kitchen area, and the manager was laundering protective clothing at their home. Statutory measures under the Food Act 2014 were taken to address food safety concerns: improvement notices in June 2019 and November 2020 and a formal warning in September 2019. The premises closed during the New Zealand government-mandated COVID-19 lockdown periods in 2020 and 2021 (Figure 1).

No CPE was detected from surface swabs or food or water specimens in July 2019 or January 2021; however, non–OXA-48–producing *E. coli* were found in multiple sites, including frequently touched kitchen surfaces such as the microwave oven door handle and keypad, cash register, and service benchtop. All specimens collected in May 2021 tested negative for CPE and *E. coli*.

Stool specimens were obtained from 16 of 18 food handlers working in May 2019. OXA-48–producing *E. coli* was detected in 4 food handlers, 2 of whom were not residents in Hutt Valley health district; 1 had a history of travel to Thailand in 2017 but had no healthcare interaction abroad. All were asymptomatic, and none were treated with antibiotic drugs. Two had HVH admission history, neither concurrent with case-patients; 1 person had undergone an outpatient procedure in a procedure room in which a case-patient was treated 4 days previously. After further testing in December 2020 (now of 11 staff members), 1 staff member had OXA-48–producing *E. coli*; this person was 1 of only 3 who had been working at the premises in 2019 and had returned a positive test at that time.

### Microbiology

We identified a total of 48 CPE-producing organisms, 41 from the 25 case-patients and 7 from the 4 food handlers. All isolates were *E. coli* and possessed OXA-48 carbapenemase and CTX-M-174 ESBL genes. They were phenotypically resistant to ceftriaxone, aztreonam, ciprofloxacin, and sulfamethoxazole/trimethoprim and were susceptible to gentamicin, amikacin, nitrofurantoin, fosfomycin, mecillinam, colistin, and meropenem.

### WGS

We sequenced and analyzed 33 isolates, with >1 from each case and each food handler (28 from case-patients and 5 from food handlers). The 33 draft genomes had a median total length of 5.03 Mb (interquartile range [IQR] 5.02–5.07 Mb; range 4.91–5.19 Mb), a median GC content of 50.8% (range 50.7%–50.8%), and a median N50 statistic of 138.65 kb (IQR 116.48–156.84 kb; range 69.37–183.45 kb). We characterized all 33 genomes as ST131 clade C. For context, we compared those 33 genomes against 12,185 ST131 clade C genome assemblies generated from publicly available sequence data (Appendix Figure 1, panel A). This analysis identified a cluster of 55 genomes. The genetic element *bla*_OXA-48_ was located on a 7,872 bp Col-type plasmid (GenBank accession no. CP175693) (Appendix Table 5).

Further comparison with an already established dataset (*23*) confirmed that the 55-genome sublineage belongs to the ST131 clade C1/*H*30R sublineage (Appendix Figure 1, panel B). The 33 genomes formed a monophyletic cluster (Appendix Figure 2, panel A), with an observed median pairwise single-nucleotide variant (SNV) distance of 7 (IQR 4–12; range 0–56) SNVs. The phylogenetic analysis demonstrates that those 33 genomes share a common ancestor with the clinical strain Camb6978 (National Center for Biotechnology Institute Sequence Read Archive [https://www.ncbi.nlm.nih.gov/sra] BioProject no. ERR2538552), which was cultivated in 2016 from a patient with a bloodstream infection in Cambodia (*24*).

**Figure 2:**
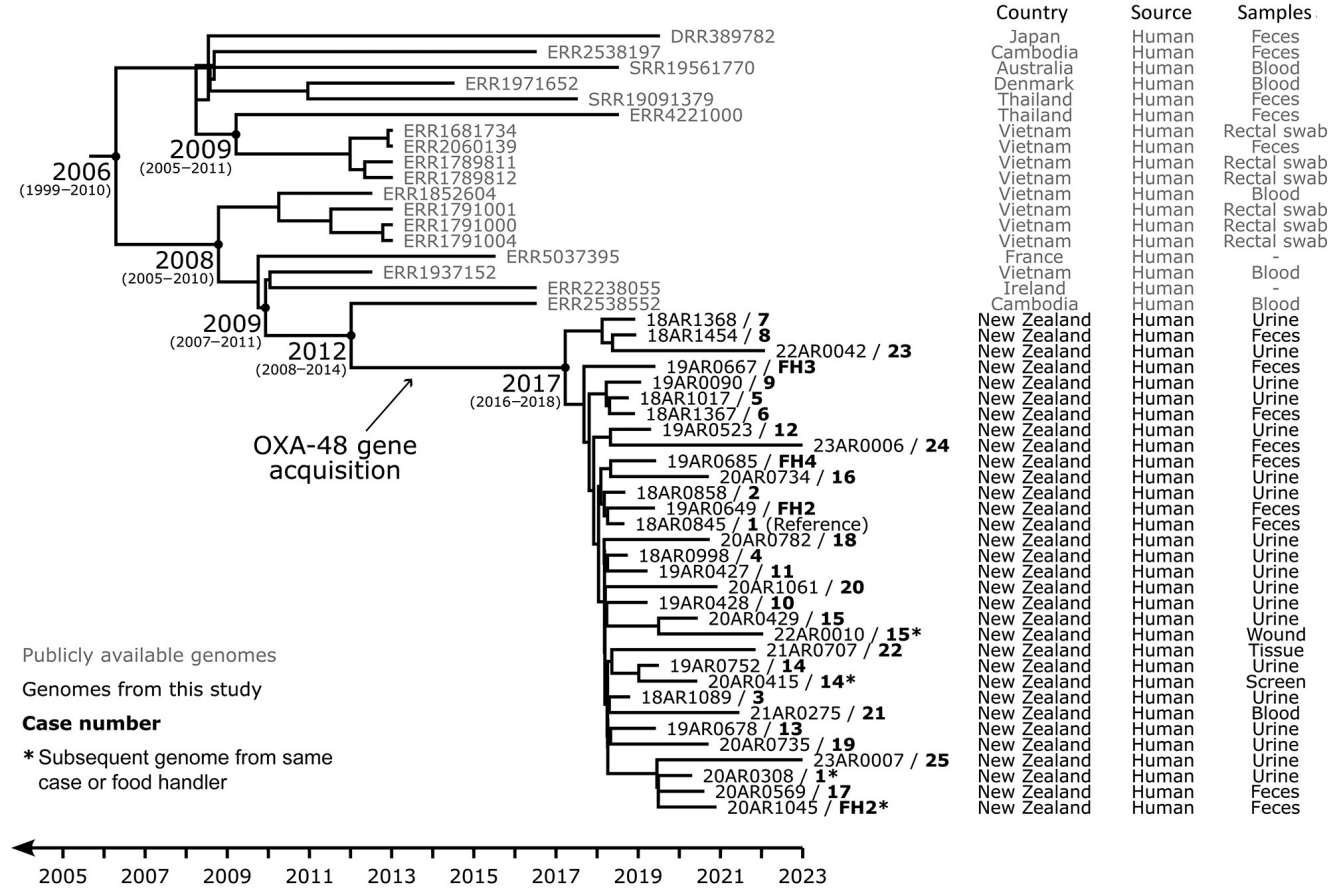
Evolutionary reconstruction for OXA-48–producing *Escherichia coli* sequence type (ST)131 genomes obtained from cases and food handlers compared with publicly available genomes in study of community outbreak linked to food premises, Hutt Valley, New Zealand, August 2018–December 2022. A time-calibrated maximum clade credibility tree was inferred from 323 nonrecombinant orthologous biallelic core-genome single-nucleotide variants (SNVs) from 50 ST131 genomes. SNVs were derived from a core-genome alignment of ≈4,767,900 bp and were called against the chromosome of 18AR0845 (GenBank accession no. CP175691). The x-axis represents the emergence time estimates. Case numbers (1–25), shown in bold after the genome codes, correspond to case reference numbers shown in Appendix Table 2). Case numbers FH1–4 indicate genomes obtained from food handlers working at a community-based food premises to which 18 of the case-patients had been exposed. Asterisks indicate subsequent genomes obtained from the same case-patient or food handler.

The outbreak isolates (n = 33) were closely related to ST131 genomes from other countries, predominantly Asia (n = 14/50, 28%) (Figure 2). Of note, genomes from Vietnam collected during 2012–2013 (*25*) appear to represent the earliest detections of this lineage. This lineage has since spread globally; representatives have been detected in Denmark (2014), France (2015 and 2018), Cambodia (2016), Ireland (2016), Thailand (2017), Australia (2018), Japan (2019), and now New Zealand (2018–2023). Screening isolates from food handlers cluster with other outbreak-associated genomes, underscoring their potential role in the dissemination of this outbreak strain (Figure 2). Furthermore, the other publicly available genomes lack the OXA-48 gene, indicating that the acquisition of this critical resistance gene likely occurred within the lineage between 2008 and 2018 (based on the 95% highest posterior density of key nodes). Evolutionary modeling estimates the cluster emerged during 2016–2018 (Appendix).

## Discussion

We report a cluster of 25 patients with an OXA-48–producing ST131 *E. coli* detected from hospital and community specimens. The occurrence of a cluster of this magnitude was unprecedented in our district: during 2009–2017 in the Wellington region (of which Hutt Valley health district is part), 14 patients had been detected with CPE, only 3 of whom had OXA-48–producing Enterobacterales (K. Dyet, unpub. data). Our investigation suggests that the cluster was at least partially linked to a community-based food premises and that transmission from colonized food handlers to customers is a likely explanation.

In total, 4 food handlers found to be colonized with the outbreak strain were working at the premises; 1 was still colonized 18 months later. Concern around food as a vector for community CPE transmission has focused on food production (*26*); a complex interplay of influences includes veterinary antibiotic use and wildlife and environmental reservoirs (*27*). However, contamination from colonized food handlers is a plausible route of spread to ready-to-eat foods. In high-prevalence settings, food handlers are not uncommonly found to be CPE carriers (*28*), and highly dynamic patterns of colonization and recolonization are also not uncommon (*29*). *E. coli* transmission in food preparation environments linked to colonized food handlers has been demonstrated in community outbreaks of enteroaggregative, enterotoxigenic, and Shiga-toxigenic *E. coli* (*30*–*32*). A foodborne outbreak of ESBL-producing *Klebsiella pneumoniae* arising from hospital-prepared food detected the outbreak strain in specimens from kitchen workers, food preparation surfaces, and food items; although the role of the kitchen staff in propagating the outbreak was unclear, evidence indicated that contaminated food was the vehicle for transmission (*33*). The outbreak reported in this study occurred in a population in which community CPE carriage is likely very rare (*7*). Detecting multiple cases within a relatively short period was highly unusual, which led to the subsequent investigation and source identification. This timing poses questions around how often foodborne spread of antimicrobial resistance mechanisms occurs but goes unnoticed in populations in which baseline community prevalence is higher.

Although our data suggest that the outbreak lineage likely originated abroad, possibly in Asia, the role of food handlers in importing the strain to New Zealand remains uncertain. One colonized food handler had traveled to Southeast Asia, but the sequencing data cannot definitively link that person’s isolate to the introduction of the outbreak strain. Culturing and sequencing of samples from food handlers was conducted 9 months after the outbreak detection in August 2018; continuing bacteria evolution in this interval meant that the 2019 samples might not perfectly represent the strain initiating the outbreak. This factor highlights the challenge of linking transmission events retrospectively when there are delays in sampling and sequencing.

Poor hand hygiene practices are often identified in outbreaks from contaminated ready-to-eat food (*31*,*32*), and hand hygiene faults occur frequently (*34*). Among food handlers in long-term care facilities, hand cleanliness was negatively correlated with *E. coli* on food contact surfaces (*35*); pathogens on hands are less likely if gloves are worn, but hygiene advantages are lost without regular glove changes and hand hygiene practices (*36*). In this outbreak, numerous food safety concerns were noted in the food premises, particularly hand hygiene practices and improper glove use, and *E. coli* was detected on food contact kitchen surfaces and high-touch points; although CPE was not detected, multiple possible food contamination pathways were present. Spread through other premise facilities (such as the shared toilets) was also possible, although CPE was not detected by testing. Use of toilets has been linked to CPE spread in healthcare environments independent of healthcare workers or person-to-person contact, including in a residential care home (*37*) and a hematological ward (*38*).

All case-patients without history of visiting premises A had been admitted to HVH; their CPE acquisition might have occurred through exposure to carriers in hospital or in the community. Nosocomial CPE transmission between patients with healthcare workers acting as possible intermediaries has been demonstrated previously in hospital outbreaks (*39*,*40*). Individual examples of community intrahousehold CPE transmission exist (*15*) but appear to be uncommon (*14*); in contrast, ESBL cocarriage and definite household transmission appears relatively frequent (*41*,*42*). Given the often-incidental case detection, further undetected carriers in the community might have been sources of transmission; however, the existence of a large pool of undetected community CPE carriage was not uncovered through enhanced community surveillance of routinely collected samples from residents from the area where case-patients lived.

The degree to which our findings can be generalized is limited. Case detection was often incidental; demographics and other case characteristics therefore skewed toward groups with higher frequency of hospital visits or higher likelihood of testing for urinary tract infection. Onset of case colonization or infection was unknowable; case exposure periods were therefore wide and approximate, potentially affecting accuracy of case-patients’ exposure recollections. CPE was not detected in food, and so our assumption of a foodborne transmission pathway relies on circumstantial observations. We did not measure the epidemiologic association with premises A with an analytical study because of difficulties inherent in recruiting representative community controls willing to be tested for CPE colonization. Finally, the outbreak was likely larger than the number of detected cases, potentially because this organism was carbapenem-susceptible and so did not always grow reliably on standard CPE screening media (Appendix).

This outbreak raises the possible role of community food premises as a source of CPE transmission. It also demonstrates challenges with controlling community CPE spread. The justification for applying traditional individually focused public health communicable disease control measures (e.g., case restriction, identification and management of contacts) is weak in a context in which short-term health risk to ambulatory colonized persons is marginal, yet the long-term public health consequences from widespread CPE spread could be formidable. CPE colonization is not a notifiable health condition in New Zealand, limiting public health action to investigate and control spread. Those constraints are not peculiar to our context; guidelines for CPE control from other jurisdictions (*4*,*43*) are primarily oriented toward the healthcare sector, and community control focuses on antibiotic stewardship. CPE poses a daunting threat to the continued effectiveness of antibiotic treatment of gram-negative infections, and a greater understanding of the epidemiology of CPE in the community is required to develop comprehensive control strategies.

Appendix Additional information about community outbreak of OXA-48–producing *Escherichia coli* linked to food premises, New Zealand, 2018–2022
