# Supplementary material for: Community Outbreak of OXA-48–Producing Escherichia coli Linked to Food Premises, New Zealand, 2018–2022
Source: Emerg Infect Dis. 2025 Jul;31(7):1300–8. doi: 10.3201/eid3107.250289 (PMC12205444; doi:10.3201/eid3107.250289)
Supplement: Appendix — Additional information about community outbreak of OXA-48–producing Escherichia coli linked to food premises, New Zealand, 2018–2022 [file 25-0289-Techapp-s1.pdf]

Article DOI: https://doi.org/10.3201/eid3107.250289

EID cannot ensure accessibility for supplementary materials supplied by authors. Readers who have difficulty accessing supplementary content should contact the authors for assistance.

Community Outbreak of OXA-48–Producing *Escherichia coli* Linked to Food Premises, New Zealand, 2018–2022

Appendix

Methods

Methods for testing clinical samples for Carbapenemase-Producing Enterobacterales

The following standard surveillance protocols for Carbapenemase-Producing Enterobacterales (CPE) testing of routinely collected clinical specimens were in place continuously through the outbreak period. Passive, or reflex, surveillance for CPE was performed on all diarrheal (loose or liquid) stool samples received from hospitalized patients if the patient had been an inpatient for >3 days or *Clostridioides difficile* testing was requested on the sample. In addition, active surveillance via rectal swab or stool sample was performed for all inpatients at if there was a history of overseas hospitalization or travel to higher-prevalence countries in the 12 months before hospital admission. Stool and rectal swab samples were plated directly onto mSuperCARBA agar (CHROMagar, St.-Denis, Ile-de-France) and incubated for 24–48 hours at 37°C in air, with suspect colonies followed up. Organisms are identified using the Vitek® MS (bioMérieux, Marcy-l'Etoile, France) with screening of the organism for carbapenemase production as per European Committee on Antimicrobial Susceptibility Testing (EUCAST) guidelines (*1*) using a 10µg meropenem disc and a 30µg temocillin disc. Organisms screened as possible carbapenemase producers had a modified carbapenem inactivation method (mCIM) test performed (*2*), and if positive a PCR (Cepheid Xpert® Carba-R, Cepheid, Sunnyvale, California, United States) to confirm the presence of one of the key carbapenemase genes. Stool samples collected for patients in the community and submitted for routine

microbiological testing were not tested for presence of CPE, other than in the enhanced community surveillance program described below.

Passive, or reflex, CPE surveillance was also performed on all urine samples from hospitalized patients when these samples were submitted for microbiological testing. Urine samples from hospital patients were plated onto a split agar plate with half Orientation agar (CHROMagar, St.-Denis, Ile-de-France) and half-blood agar with 1% aztreonam. All *E. coli* growing on the 1% aztreonam agar (regardless of growth quantity) were followed up for resistance mechanism determination, which included screening for carbapenemase production using the same steps as outlined for stool samples. The same process was followed with urine samples from patients in the community, however resistance mechanism determination only occurred if clinically-significant cultures were identified (unless part of the enhanced community surveillance program).

Active case finding was initiated when a patient is detected with CPE either during or immediately following a hospital inpatient episode, and either there was suspicion of acquisition on the ward or the patient was not managed in appropriate transmission-based precautions during their admission. This is undertaken by identifying patients who shared the index patient's ward room or toilet facilities (contacts) and screening by testing either a stool specimen or rectal swab for presence of CPE at least 7 days after last contact. A modified screening method was used to enhance sensitivity for the outbreak OXA-48-producing *E. coli* in active case finding among contacts of hospitalized patients detected with this organism. This was required because the organism was carbapenem susceptible and therefore did not grow consistently on the standard screening agar. To overcome this, an ESBL agar was used: stools were plated directly onto ESBL/Vancomycin-resistant Enterococcus (VRE) chromogenic agar (CHROMagar, St.-Denis, Ile-de-France), with suspect colonies followed up, as per the manufacturer's instructions. An ESBL agar was chosen because the target organism was known to possess an ESBL enzyme. Suspect organisms were further identified as described above for reflex surveillance of stool samples. This method was also used for the testing of stool samples submitted by food handlers.

A fixed-term enhanced community surveillance program was undertaken over an 8-month period from 2020 to 2021 to detect additional community cases with OXA-48-producing *E. coli* fecal carriage or bacteriuria. The program was conducted on samples collected from

patients aged over 16 years of age and residing in a target area of Hutt Valley comprising nine contiguous suburbs, with a resident population of ≈38,000 (*3*), in which 64% of the known community cases were living when diagnosed. In this program, the laboratory thresholds applied for CPE screening were lower than the standard thresholds described above. In the enhanced community surveillance program, screening for CPE was conducted on all consecutive community stool samples and on all consecutive urine samples received for microbiology testing during the surveillance. Enhanced community surveillance stool samples were tested with the process described above for active case finding among hospital contacts, with initial plating of stool onto ESBL/VRE agar. Enhanced community surveillance urine samples were tested as for urine samples collected from hospitalized patients.

Sampling and testing of environmental specimens from the food premises

A sampling scheme was developed before premises visit and used to guide sample collection. Sampling was directed at frequently touched surfaces and objects in the kitchen, including benches, cupboard handles, kitchen equipment buttons, and handles. Sink drains were sampled inside the downpipes. Toilets were sampled under the rim and across the top of toilet seat. Samples were collected from the inside of touch-free hand dryers.

Flocked swabs were used for sampling environmental surfaces and objects. Swabs were used as is if sampling wet sites and pre-moistened with sterile saline if used for sampling a dry site. Flocked swabs were packaged with Liquid Amies for transportation. Specimens were transported promptly to the laboratory for immediate processing.

The samples were screened for CPE following the methods given in the *Victorian guideline on environmental sampling for carbapenemase-producing Enterobacteriaceae, Version 1* (*4*). All samples were enriched in trypticase soy broth (TSB): swabs were enriched in 20 mL TSB; 25 g of food samples was homogenized with 225 mL TSB to make a 1:10 dilution; and for water samples 20 mL of TSB was added to the sample. For all samples, incubation with TSB was at 35°C for 48 hours and then the broth was checked for turbidity. If turbid, 10 µL of the broth was streaked onto a CHROMagar ESBL plate which was incubated at 37°C for 24 hours. If the TSB was not turbid after 48 hours, it was reincubated for a further 24 hours and checked again, and streaked out as above if turbid. Colonies suspected of being extended spectrum β-lactamase (ESBL) producing *E. coli* were sent to the Institute of Environmental

Research (ESR) Antimicrobial Resistance Laboratory and screened for carbapenemase production using the current Clinical and Laboratory Standards Institute (CLSI) modified carbapenem inactivation method (CLSI supplement M100). The samples were also tested for the presence of generic *E. coli* by enrichment in lactose broth followed by detection in EC-MUG broth.

Confirmatory testing and whole-genome sequencing

Illumina library construction and Illumina sequencing

As part of the ESR national surveillance of Enterobacterales with acquired carbapenemases (*5*), all CPE were characterized using whole-genome sequencing (WGS). CPE cultures were plated on trypticase soy agar and incubated at 35°C for 18 hours. Following incubation, the cultures were examined for viability and purity. A single colony was subcultured and used for WGS. DNA was extracted using either the Roche High Pure PCR template preparation kit (Roche Diagnostics, Mannheim, Germany) or the chemagic 360 (PerkinElmer Inc., Waltham, Massachusetts, United States). The DNA library was created using the Nextera XT DNA preparation kit (Illumina Inc., San Diego, California, United States) or the PlexWell Library Preparation kit (seqWell, Boston, Massachusetts, United States), and sequenced as 2×151 bp paired-end reads on the NextSeq 550 platform using V2.5 chemistry (Illumina) at ESR (Kenepuru, Porirua, New Zealand).

Illumina sequencing data quality control and de novo assembly

Raw reads were checked for quality using FastQC v0.12.1 (http://www.bioinformatics.babraham.ac.uk/projects/fastqc/, accessed on 23 November 2024). To perform taxonomic profiling and detect *E. coli* in the raw Illumina sequence data, we used Kraken v2.1.3 (*6*) with default parameters and an NCBI Reference Sequence (RefSeq) database (*7*), Standard (https://benlangmead.github.io/aws-indexes/k2, accessed on 05 June 2023). The database contained references for archaea, bacteria, human, viruses, plasmids, and the 'UniVec core' subset of the UniVec database (a database of vector, adaptor, linker, and primer sequences).

Raw sequence reads were de novo assembled using Shovill v1.1.0 (https://github.com/tseemann/shovill, accessed on 22 November 2024), which utilizes: Seqtk v1.3-r106 (https://github.com/lh3/seqtk, accessed on 22 November 2024); Trimmomatic v0.36

(*8*); Lighter v1.1.2 (*9*); FLASH v1.2.11 (*10*); SKESA v2.4.0 (*11*,*12*); Samclip v0.4.0 (https://github.com/tseemann/samclip, 22 November 2024); SAMtools v1.16.1 (*13*), the Burrows-Wheeler Aligner (BWA) v0.7.17 (*14*); and Pilon v1.24 (*15*). Shovill was used with parameters set to: (i) estimate the genome size to 4.6 Mb; (ii) remove contiguous sequences (contigs) with a sequence coverage below 20-fold; and (iii) enable single-cell mode. Assembly metrics were assessed using QUAST v5.0.2 (*16*). The Illumina sequence data quality metrics were assessed and are outlined in Appendix Table 4.

In silico genotyping of Illumina assemblies

In silico multilocus sequence typing (MLST) was done using MLST v2.23.0 (https://github.com/tseemann/mlst, accessed on 22 November 2024) with default settings to query the assemblies against the Achtman *E. coli* MLST typing database hosted on PubMLST (*17*,*18*). ABRicate v1.0.1 (https://github.com/tseemann/abricate, accessed on 22 November 2024) was used to screen the assemblies for acquired antibiotic resistance genes using the ARG-ANNOT (*19*) database (last updated 15 September 2023).

Nanopore sequencing

DNA libraries were prepared using the ONT gDNA rapid barcoding kit 96 (SQK-RBK0004, Oxford, United Kingdom) as per the manufacturer's instructions. The entire library was loaded onto an R9.4.1 flow cell (FLO-MIN106) and run on a MinION device for ≈20 to 40 hours (using MinKNOW versions 0.45.2.6–2.34.3).

Generating a reference genome for 18AR0845

The reads generated from the MinION sequencing run were basecalled using the Oxford Nanopore Technologies Albacore v2.3.1. NanoStat v1.6.0 from Nanopack v1.6.0 (*20*) was used to perform an initial quality assessment on the raw nanopore reads. Additionally, NanoQC v0.9.4 from NanoPack was used to assess the overall quality of the sequencing data. NanoFilt v2.8.0, also from NanoPack, was used for read trimming. Initially, 52 nt were trimmed from the start and end of each read to remove low-quality regions from the reads. Subsequently, NanoFilt was used again to filter out reads with a quality score below Q7. To perform taxonomic profiling and detect *E. coli* in the trimmed and filtered nanopore sequence data, we used Kraken2 as described above (single-ended read mode).

The trimmed and filtered nanopore reads were assembled de novo using Flye v2.7 (*21*,*22*), with the genome size estimated at 4.6 Mb and three polishing iterations. The assembly underwent three rounds of additional polishing by mapping the corresponding nanopore reads to each contig using minimap2 v2.24 (*23*,*24*), and then correcting single nucleotide variants (SNVs) and insertions and deletions (INDELs) with racon v1.4.3 (*25*) with parameters: '–match 8' for match score, '–mismatch −6' for mismatch score, and '–gap −8' for gap penalty. After using the nanopore reads to polish with racon, the assemblies were further refined using medaka v1.11.3 (https://github.com/nanoporetech/medaka, accessed on 05 December 2024), using the 'super accuracy' model r941_min_sup_g507. The chromosome was reoriented to start at the *dna*A gene using Circlator v1.5.1 (*26*). The chromosome and each plasmid assembly then underwent five rounds of additional polishing by mapping the corresponding Illumina reads to each contig using BWA-MEM and then correcting SNVs and small INDELs with Pilon v1.24 (*15*).

ABRicate was used to screen the complete genome of 18AR0845 for O and H-antigens using the EcOH database (*27*) (last updated 15 September 2023). The *fim*H allele was characterized using and FimTyper 1.0 (https://cge.food.dtu.dk/services/FimTyper/, accessed 05 December 2024) with default parameters. Virulence genes, acquired antibiotic resistance genes, and mutations conferring resistance to antibiotics were identified using AMRfinderplus v3.12.8 with database version 2024–01–31.1 (*28*). The assembly was annotated using Prokka v1.14.6 (*29*). Prophage regions were identified using PHASTER (*30*) and then annotated using Pharokka v1.6.1 (*31*). Mobile genetic elements were identified using IslandViewer 4 (*32*) and ISsaga v2.0 (*33*) (ISfinder platform (*34*)), followed by manual curation using Artemis v18.2.0 (*35*). Summary metrics are reported in Appendix Table 5.

Dataset curation with additional publicly available genomes

To identify additional sequence type (ST)131 genomes relating to this cluster, we first screened the Enterobase database v1.2.0 (https://enterobase.warwick.ac.uk/, accessed 20 November 2023) for genomes belonging to ST131 based on the Achtman scheme (i.e., *adk*, *fum*C, *gyr*B, *icd*, *mdh*, *pur*A, and *rec*A). This screening identified 16,327 ST131 genomes. We retrieved the sequence read data for these 16,327 ST131 genomes from the National Center for Biotechnology Information (NCBI) sequence read archive (SRA) using the 'fasterq-dump' tool within the SRA Toolkit v3.0.1-ubuntu64 (https://github.com/ncbi/sra-tools, accessed 22

November 2023). Raw sequence reads were de novo assembled using the Shovill pipeline (as described above).

Assembly-based variant detection and initial ST131 phylogenetic analyses

A total of 16,360 *E. coli* ST131 genome assemblies (33 sequenced in this study and 16,327 from Enterobase) were aligned to create a core-genome alignment using Parsnp v1.7.4 (*36*), with the reference being the chromosome of EC958 (GenBank: HG941718), to identify SNVs. Resulting SNV alignments were used to reconstruct phylogenies. RaxML v8.2.12 (*37*) built phylogenetic trees using the maximum-likelihood method with GTR-GAMMA correction (optimising 10 distinct, randomized maximum-parsimony trees before adding 1,000 bootstrap replicates). The phylogenetic trees were visualized using FigTree v1.4.4 (http://tree.bio.ed.ac.uk/software/figtree/, accessed 11 November 2024). Among the 16,327 publicly available genomes, 12,185 (74.6%) were identified as belonging to Clade C. To enhance clarity, we repeated the analysis using only Clade C genomes, subsequently placing the sub-lineage of interest into the context of the previously reported ST131 lineage (*38*).

High-resolution cluster phylogeny

Our dataset for the ST131 Clade C1/*H*30R sub-lineage consisted of 55 genomes (Appendix Table 1 and Appendix Table 6). The SPANDx v4.0.4 pipeline (*39*) was used for identifying genetic variants through a read-mapping method. Briefly, Illumina short-reads were mapped to the complete 18AR0845 chromosome (GenBank: CP175691). SNVs within regions of high-density clusters (≥3 SNVs found within a 10 bp window), mobile genetic elements, and predicted recombination sites (identified using Gubbins v3.3.5 (*40*)) were removed from the core-genome alignment (Appendix Table 7). Sites were excluded if a SNV was called in regions with less than half or greater than 3-fold the average genome coverage on a genome-by-genome basis. This analysis defines a core genome as regions estimated to the nearest 100 bp with ≥95% coverage across one or more genomes in the given population. The pairwise SNV distances were determined using snp-dist v0.6.3 (https://github.com/tseemann/snp-dists, accessed on 11 November 2024). A maximum-parsimony tree was reconstructed from the orthologous biallelic core-genome SNV alignment using the heuristic search feature of PAUP v4.0a (*41*). The resulting phylogenetic trees were visualized using FigTree.

Bayesian phylogenetic analysis using BEAST2

Bayesian phylogenetic analysis was performed using BEAST2 v2.7.7 (*42*,*43*), following the approach described in our previous study (*44*). Tip-dating methods were applied using TempEst v1.5.3 (*45*) to identify an initial clock rate (6.17×10^{-3} substitutions per site per year). This was input into BEAUTI v2.7.7, with a uniform prior and an upper bound of 0.1. All other priors were left as default. The Gamma Site Model Category Count was set to four, and the GTR substitution model rates, determined from jModelTest v2.1.10 (*46*), were included (AC = 0.90, AG = 4.26, AT = 1.35, CG = 0.14, CT = 4.13, GT = 1.00). To test if the strict clock or uncorrelated relaxed clock best fits our dataset, initial models were created using tip dates, a GTR substitution model, and a coalescent prior with a constant population. Both models were tested with the Nested sampling Bayesian computation algorithm v1.1.0 within the BEAST2 package (particle count: 32, sub-chain length: 5000, Epsilon: 1.0×10^{-12}).

Various population models were compared to ensure the selection of the best-fit model. Three models were tested with the relaxed log-normal clock model, including the Bayesian skyline, coalescent constant, and exponential growth population size models. Once the best-fitting model was identified, three independent Markov chain Monte Carlo (MCMC) runs of 100 million generations each were performed, sampling every 1,000 generations. Outputs were assessed in Tracer v1.7.2 (http://github.com/beast-dev/tracer/, accessed on 25 November 2024) for convergence, and replicate runs were combined in LogCombiner v2.7.7 (BEAST2 package) with a 10% burn-in. Maximum clade credibility trees were generated in TreeAnnotator v2.7.7 (BEAST2 package), and the final phylogenies were visualized in FigTree. Further methodological details are available in our previous publication (*44*).

Results

Estimation of cluster emergence date

We used the root-to-tip feature in TempEst to estimate the temporal placement of the phylogenetic tips, followed by the more computationally intensive BEAST2. First, a maximum likelihood tree of 55 genomes, constructed using 602 core-genome SNVs, served as the input for TempEst (Appendix Figure 2a). Five genomes (DRR387864, DRR389827, SRR5936518, ERR5037306, 19AR0650/FH1) were excluded from the TempEst analysis due to the root-to-tip

divergence falling outside the predicted interval, likely due to possible errors in the sequence data or sample metadata. Consequently, the phylogeny was reconstructed with the remaining 50 genomes, using an alignment of 323 core-genome SNVs (Appendix Figure 2b). The sub-lineage of interest exhibited a linear relationship between divergence time and evolutionary distance (correlation coefficient = 0.82). Regression analysis in TempEst estimated a mutation rate of 6.17×10^{-3} substitutions per site ($R^2 = 0.68$) (Appendix Figure 2b). The time to the most recent common ancestor (MRCA) is estimated at the end of 2006 (95% confidence interval: 2003 to 2009).

After confirming an appropriate temporal signal in the dataset (n = 50), the Nested Sampling Bayesian algorithm identified the uncorrelated relaxed log-normal clock model as the better fit. The marginal likelihood estimate for the relaxed clock model was −2,370.05 (±2.00), compared to −2,376.63 (±1.84) for the strict clock model. Using the Bayesian skyline population size change model (Appendix Table 8), BEAST2 pinpointed the time to MRCA to 2006 (95% highest posterior density (HPD): 1999 to 2010) (Figure 2 in main article) (based on median node height) and estimated median mutation rate of 6.46×10^{-3} substitutions per site per year (95% HPD: 4.41×10^{-3} to 8.91×10^{-3}). To address ascertainment bias, the dataset describes one SNV for every 14,761.3 bases across the ~4.8 Mb core-genome, yielding a genome-wide mutation rate of 4.38×10^{-7} mutations/year/site. This aligns with consistent with prior estimates for *E. coli* (4.14×10^{-7} to 6.73×10^{-7}) (*47–50*), and *Shigella* (6.0×10^{-7}) (*51*). This corresponds to 2.1 fixated SNVs per year per genome (95% HPD: 0.1 to 2.9), meaning isolates sharing an MRCA 1 year prior would typically differ by 0 to 6 SNVs.

References

1. European Committee on Antimicrobial Susceptibility Testing (EUCAST). EUCAST guidelines for detection of resistance mechanisms and specific resistances of clinical and/or epidemiological importance. Version 2.0, July 2017. European Society of Clinical Microbiology and Infectious Diseases; 2017. http://www.eucast.org/resistance_mechanisms

2. Pierce VM, Simner PJ, Lonsway DR, Roe-Carpenter DE, Johnson JK, Brasso WB, et al. Modified carbapenem inactivation method for phenotypic detection of carbapenemase production among *Enterobacteriaceae.* J Clin Microbiol. 2017;55:2321–33. PubMed https://doi.org/10.1128/JCM.00193-17

3. Stats NZ Tatauranga Aotearoa. 2018 Census place summaries. [cited 2024 Aug 21]. https://www.stats.govt.nz/tools/2018-census-place-summaries/.

4. State of Victoria, Department of Health and Human Services. Victorian guideline on environmental sampling for carbapenemase-Producing Enterobacteriaceae. Version 1. Melbourne (Vic): Victorian Government; 2018. https://www2.health.vic.gov.au/public-health/infectious-diseases/infection-control-guidelines/carbapenemase-producing-enterobacteriaceae-management

5. Institute of Environmental Science and Research. Enterobacterales with acquired carbapenemases, 2021. [cited 2024 Aug 7]. https://www.esr.cri.nz/digital-library/2021-enterobacterales-with-acquired-carbapenemases/

6. Wood DE, Salzberg SL. Kraken: ultrafast metagenomic sequence classification using exact alignments. Genome Biol. 2014;15:R46. PubMed https://doi.org/10.1186/gb-2014-15-3-r46

7. Sayers EW, Barrett T, Benson DA, Bryant SH, Canese K, Chetvernin V, et al. Database resources of the National Center for Biotechnology Information. Nucleic Acids Res. 2009;37(Database):D5–15. PubMed https://doi.org/10.1093/nar/gkn741

8. Bolger AM, Lohse M, Usadel B. Trimmomatic: a flexible trimmer for Illumina sequence data. Bioinformatics. 2014;30:2114–20. PubMed https://doi.org/10.1093/bioinformatics/btu170

9. Song L, Florea L, Langmead B. Lighter: fast and memory-efficient sequencing error correction without counting. Genome Biol. 2014;15:509. PubMed https://doi.org/10.1186/s13059-014-0509-9

10. Magoč T, Salzberg SL. FLASH: fast length adjustment of short reads to improve genome assemblies. Bioinformatics. 2011;27:2957–63. PubMed https://doi.org/10.1093/bioinformatics/btr507

11. Souvorov A, Agarwala R. SAUTE: sequence assembly using target enrichment. BMC Bioinformatics. 2021;22:375. PubMed https://doi.org/10.1186/s12859-021-04174-9

12. Souvorov A, Agarwala R, Lipman DJ. SKESA: strategic k-mer extension for scrupulous assemblies. Genome Biol. 2018;19:153. PubMed https://doi.org/10.1186/s13059-018-1540-z

13. Li H, Handsaker B, Wysoker A, Fennell T, Ruan J, Homer N, et al.; 1000 Genome Project Data Processing Subgroup. The Sequence Alignment/Map format and SAMtools. Bioinformatics. 2009;25:2078–9. PubMed https://doi.org/10.1093/bioinformatics/btp352

14. Li H, Durbin R. Fast and accurate short read alignment with Burrows-Wheeler transform. Bioinformatics. 2009;25:1754–60. PubMed https://doi.org/10.1093/bioinformatics/btp324

15. Walker BJ, Abeel T, Shea T, Priest M, Abouelliel A, Sakthikumar S, et al. Pilon: an integrated tool for comprehensive microbial variant detection and genome assembly improvement. PLoS One. 2014;9:e112963. PubMed https://doi.org/10.1371/journal.pone.0112963

16. Gurevich A, Saveliev V, Vyahhi N, Tesler G. QUAST: quality assessment tool for genome assemblies. Bioinformatics. 2013;29:1072–5. PubMed https://doi.org/10.1093/bioinformatics/btt086

17. Wirth T, Falush D, Lan R, Colles F, Mensa P, Wieler LH, et al. Sex and virulence in *Escherichia coli*: an evolutionary perspective. Mol Microbiol. 2006;60:1136–51. PubMed https://doi.org/10.1111/j.1365-2958.2006.05172.x

18. Larsen MV, Cosentino S, Rasmussen S, Friis C, Hasman H, Marvig RL, et al. Multilocus sequence typing of total-genome-sequenced bacteria. J Clin Microbiol. 2012;50:1355–61. PubMed https://doi.org/10.1128/JCM.06094-11

19. Gupta SK, Padmanabhan BR, Diene SM, Lopez-Rojas R, Kempf M, Landraud L, et al. ARG-ANNOT, a new bioinformatic tool to discover antibiotic resistance genes in bacterial genomes. Antimicrob Agents Chemother. 2014;58:212–20. PubMed https://doi.org/10.1128/AAC.01310-13

20. De Coster W, D'Hert S, Schultz DT, Cruts M, Van Broeckhoven C. NanoPack: visualizing and processing long-read sequencing data. Bioinformatics. 2018;34:2666–9. PubMed https://doi.org/10.1093/bioinformatics/bty149

21. Lin Y, Yuan J, Kolmogorov M, Shen MW, Chaisson M, Pevzner PA. Assembly of long error-prone reads using de Bruijn graphs. Proc Natl Acad Sci U S A. 2016;113:E8396–405. PubMed https://doi.org/10.1073/pnas.1604560113

22. Kolmogorov M, Yuan J, Lin Y, Pevzner PA. Assembly of long, error-prone reads using repeat graphs. Nat Biotechnol. 2019;37:540–6. PubMed https://doi.org/10.1038/s41587-019-0072-8

23. Li H. Minimap2: pairwise alignment for nucleotide sequences. Bioinformatics. 2018;34:3094–100. PubMed https://doi.org/10.1093/bioinformatics/bty191

24. Li H. New strategies to improve minimap2 alignment accuracy. Bioinformatics. 2021;37:4572–4. PubMed https://doi.org/10.1093/bioinformatics/btab705

25. Vaser R, Sović I, Nagarajan N, Šikić M. Fast and accurate de novo genome assembly from long uncorrected reads. Genome Res. 2017;27:737–46. PubMed https://doi.org/10.1101/gr.214270.116

26. Hunt M, Silva ND, Otto TD, Parkhill J, Keane JA, Harris SR. Circlator: automated circularization of genome assemblies using long sequencing reads. Genome Biol. 2015;16:294. PubMed https://doi.org/10.1186/s13059-015-0849-0

27. Ingle DJ, Valcanis M, Kuzevski A, Tauschek M, Inouye M, Stinear T, et al. *In silico* serotyping of *E. coli* from short read data identifies limited novel O-loci but extensive diversity of O:H serotype combinations within and between pathogenic lineages. Microb Genom. 2016;2:e000064. PubMed https://doi.org/10.1099/mgen.0.000064

28. Feldgarden M, Brover V, Gonzalez-Escalona N, Frye JG, Haendiges J, Haft DH, et al. AMRFinderPlus and the Reference Gene Catalog facilitate examination of the genomic links among antimicrobial resistance, stress response, and virulence. Sci Rep. 2021;11:12728. PubMed https://doi.org/10.1038/s41598-021-91456-0

29. Seemann T. Prokka: rapid prokaryotic genome annotation. Bioinformatics. 2014;30:2068–9. PubMed https://doi.org/10.1093/bioinformatics/btu153

30. Zhou Y, Liang Y, Lynch KH, Dennis JJ, Wishart DS. PHAST: a fast phage search tool. Nucleic Acids Res. 2011;39(suppl):W347-52. PubMed https://doi.org/10.1093/nar/gkr485

31. Bouras G, Nepal R, Houtak G, Psaltis AJ, Wormald PJ, Vreugde S. Pharokka: a fast scalable bacteriophage annotation tool. Bioinformatics. 2023;39:btac776. PubMed https://doi.org/10.1093/bioinformatics/btac776

32. Bertelli C, Laird MR, Williams KP, Lau BY, Hoad G, Winsor GL, et al.; Simon Fraser University Research Computing Group. IslandViewer 4: expanded prediction of genomic islands for larger-scale datasets. Nucleic Acids Res. 2017;45(W1):W30–5. PubMed https://doi.org/10.1093/nar/gkx343

33. Varani AM, Siguier P, Gourbeyre E, Charneau V, Chandler M. ISsaga is an ensemble of web-based methods for high throughput identification and semi-automatic annotation of insertion sequences in prokaryotic genomes. Genome Biol. 2011;12:R30. PubMed https://doi.org/10.1186/gb-2011-12-3-r30

34. Siguier P, Perochon J, Lestrade L, Mahillon J, Chandler M. ISfinder: the reference centre for bacterial insertion sequences. Nucleic Acids Res. 2006;34:D32–6. PubMed https://doi.org/10.1093/nar/gkj014

35. Rutherford K, Parkhill J, Crook J, Horsnell T, Rice P, Rajandream M-A, et al. Artemis: sequence visualization and annotation. Bioinformatics. 2000;16:944–5. PubMed https://doi.org/10.1093/bioinformatics/16.10.944

36. Treangen TJ, Ondov BD, Koren S, Phillippy AM. The Harvest suite for rapid core-genome alignment and visualization of thousands of intraspecific microbial genomes. Genome Biol. 2014;15:524. PubMed https://doi.org/10.1186/s13059-014-0524-x

37. Stamatakis A. RAxML version 8: a tool for phylogenetic analysis and post-analysis of large phylogenies. Bioinformatics. 2014;30:1312–3. PubMed https://doi.org/10.1093/bioinformatics/btu033

38. Kidsley AK, White RT, Beatson SA, Saputra S, Schembri MA, Gordon D, et al. Companion animals are spillover hosts of the multidrug-resistant human extraintestinal *Escherichia coli* pandemic clones ST131 and ST1193. Front Microbiol. 2020;11:1968. PubMed https://doi.org/10.3389/fmicb.2020.01968

39. Sarovich DS, Price EP. SPANDx: a genomics pipeline for comparative analysis of large haploid whole genome re-sequencing datasets. BMC Res Notes. 2014;7:618. PubMed https://doi.org/10.1186/1756-0500-7-618

40. Croucher NJ, Page AJ, Connor TR, Delaney AJ, Keane JA, Bentley SD, et al. Rapid phylogenetic analysis of large samples of recombinant bacterial whole genome sequences using Gubbins. Nucleic Acids Res. 2015;43:e15–15. PubMed https://doi.org/10.1093/nar/gku1196

41. Wilgenbusch JC, Swofford D. Inferring Evolutionary Trees with PAUP*. Curr Protoc Bioinformatics 2003 Feb:Chapter 6:Unit 6.4.

42. Bouckaert R, Heled J, Kühnert D, Vaughan T, Wu C-H, Xie D, et al. BEAST 2: a software platform for Bayesian evolutionary analysis. PLOS Comput Biol. 2014;10:e1003537. PubMed https://doi.org/10.1371/journal.pcbi.1003537

43. Bouckaert R, Vaughan TG, Barido-Sottani J, Duchêne S, Fourment M, Gavryushkina A, et al. BEAST 2.5: An advanced software platform for Bayesian evolutionary analysis. PLOS Comput Biol. 2019;15:e1006650. PubMed https://doi.org/10.1371/journal.pcbi.1006650

44. White RT, Bakker S, Burton M, Castro ML, Couldrey C, Dyet K, et al. Rapid identification and subsequent contextualization of an outbreak of methicillin-resistant *Staphylococcus aureus* in a neonatal intensive care unit using nanopore sequencing. Microb Genom. 2024;10:001273. PubMed https://doi.org/10.1099/mgen.0.001273

45. Rambaut A, Lam TT, Max Carvalho L, Pybus OG. Exploring the temporal structure of heterochronous sequences using TempEst (formerly Path-O-Gen). Virus Evol. 2016;2:vew007. PubMed https://doi.org/10.1093/ve/vew007

46. Darriba D, Taboada GL, Doallo R, Posada D. jModelTest 2: more models, new heuristics and parallel computing. Nat Methods. 2012;9:772. PubMed https://doi.org/10.1038/nmeth.2109

47. White RT, Bull MJ, Barker CR, Arnott JM, Wootton M, Jones LS, et al. Genomic epidemiology reveals geographical clustering of multidrug-resistant *Escherichia coli* ST131 associated with bacteraemia in Wales. Nat Commun. 2024;15:1371. PubMed https://doi.org/10.1038/s41467-024-45608-1

48. Kallonen T, Brodrick HJ, Harris SR, Corander J, Brown NM, Martin V, et al. Systematic longitudinal survey of invasive *Escherichia coli* in England demonstrates a stable population structure only transiently disturbed by the emergence of ST131. Genome Res. 2017;27:1437–49. PubMed https://doi.org/10.1101/gr.216606.116

49. Ben Zakour NL, Alsheikh-Hussain AS, Ashcroft MM, Khanh Nhu NT, Roberts LW, Stanton-Cook M, et al. Sequential acquisition of virulence and fluoroquinolone resistance has shaped the evolution of *Escherichia coli* ST131. mBio. 2016;7:e00347–16. PubMed

50. Ludden C, Decano AG, Jamrozy D, Pickard D, Morris D, Parkhill J, et al. Genomic surveillance of *Escherichia coli* ST131 identifies local expansion and serial replacement of subclones. Microb Genom. 2020;6:e000352. PubMed https://doi.org/10.1099/mgen.0.000352

51. Holt KE, Thieu Nga TV, Thanh DP, Vinh H, Kim DW, Vu Tra MP, et al. Tracking the establishment of local endemic populations of an emergent enteric pathogen. Proc Natl Acad Sci U S A. 2013;110:17522–7. PubMed https://doi.org/10.1073/pnas.1308632110

Appendix Table 1. Fields comprising standardized interview schedule used to gather case characteristic and potential exposure data on cases identified with OXA-48-producing *Escherichia coli*, Lower Hutt, New Zealand, August 2018 – December 2022

Interview schedule category	Interview schedule fields within category
Case demographics	Case name; national health identification number; address; ethnicity; occupation; voluntary work
Clinical history and exposures	Date of first CPE detection; illness/symptoms at time of test; usual primary heatlhcare provider; medical centers visited before diagnosis; home-based healthcare or support services used before diagnosis; use of home mobility aids; hospital, respite care or long term care facility exposure
Persons in case's household	Occupation(s); place(s) of work/school, international and domestic travel history, hospital admissions international or domestic
Close non-household contacts of case	Occupation(s); place(s) of work/school, international and domestic travel history, hospital admissions international or domestic
Travel history	International travel in prior 4 y (travel dates and countries/regions visited); illness while traveling; visits to healthcare facilities while traveling; medical treatment or procedures while traveling; travel with the intent of receiving medical, dental or other healthcare; domestic travel outside region
Food history	Usual places where groceries purchased; summary of usual grocery purchases; consumption of food from ready-to-eat food premises including restaurants, cafes, bars, takeaway food premises, markets, events, sports clubs; consumption of imported food
Drinking water and recreational water	Consumption of drinking water from non-reticulated supply; participation in swimming, kayaking or other water sports
Animal contact	Contact with animals, including domestic pets, farm animals; contact with any animals with illness
Environmental exposures	Contact with rural environments; use of public conveniences

Appendix Table 2. Exposure of 25 cases with OXA-48-producing *Escherichia coli* to a ready-to-eat food premises implicated as a possible source of transmission (Premises A), Lower Hutt, New Zealand, August 2018 – December 2022

Case reference number	Date first sample collected from which OXA-48-producing *E. coli* detected	Most recent visit to Premises A before OXA-48-producing *E. coli* detection, if known
1	Aug 2018	Aug 2018
2	Aug 2018	Jun 2018
3	Oct 2018	Aug 2018
4	Sep 2018	Visited four times in prior 6 mo; specific dates unknown
5	Sep 2018	No visit to premises
6	Nov 2018	Sep 2018
7	Nov 2018	No visit to premises
8	Dec 2018	No visit to premises
9	Jan 2019	Dec 2018
10	Mar 2019	Dec 2018
11	Mar 2019	Jan 2019
12	Apr 2019	Dec 2018
13	May 2019	Feb 2019
14	Jun 2019	No visit to premises
15	Jun 2020	Aug 2019
16	Sep 2020	Jul 2020
17	Jul 2020	Jul 2020
18	Sep 2020	Aug 2020
19	Sep 2020	Visited 3–4 times since May 2020; specific dates unknown
20	Nov 2020	Visited twice in 2019, not subsequently
21	Jun 2021	Visited premises before March 2020
22	Oct 2021	No visit to premises
23	Jan 2022	No visit to premises
24	Dec 2022	Visits to premises unknown
25	Dec 2022	Visited premises in 2018; specific dates unknown

Appendix Table 3. Whole-genome sequences of 33 *Escherichia coli* samples used in this investigation: Metadata

ESR ID	MLST	Year	Source	Age	Specimen	Bio Project ID	BioSample	Illumina SRA
18AR0845	ST131	2018	Human	90 to <95	Feces	PRJNA1102395	SAMN41036272	SRR28760499
18AR0858	ST131	2018	Human	70 to <75	Urine	PRJNA1102395	SAMN41036273	SRR28760498
18AR0998	ST131	2018	Human	55 to <60	Urine	PRJNA1102395	SAMN41036280	SRR28760490
18AR1017	ST131	2018	Human	40 to <45	Urine	PRJNA1102395	SAMN41036282	SRR28760488
18AR1089	ST131	2018	Human	80 to <85	Urine	PRJNA1102395	SAMN41036283	SRR28760487
18AR1367	ST131	2018	Human	70 to <75	Feces	PRJNA1102395	SAMN41036292	SRR28760671
18AR1368	ST131	2018	Human	85 to <90	Urine	PRJNA1102395	SAMN41036293	SRR28760670
18AR1454	ST131	2018	Human	50 to <55	Feces	PRJNA1102395	SAMN41036298	SRR28760664
19AR0090	ST131	2019	Human	90 to <95	Urine	PRJNA1102395	SAMN41036309	SRR28760652
19AR0427	ST131	2019	Human	80 to <85	Urine	PRJNA1102395	SAMN41036330	SRR28760565
19AR0428	ST131	2019	Human	80 to <85	Urine	PRJNA1102395	SAMN41036331	SRR28760564
19AR0523	ST131	2019	Human	70 to <75	Urine	PRJNA1102395	SAMN41036340	SRR28760554
19AR0649	ST131	2019	Human	55 to <60	Feces	PRJNA1102395	SAMN41036345	SRR28760774
19AR0650	ST131	2019	Human	45 to <50	Feces	PRJNA1102395	SAMN41036346	SRR28760773
19AR0667	ST131	2019	Human	25 to <30	Feces	PRJNA1102395	SAMN41036349	SRR28760769
19AR0678	ST131	2019	Human	70 to <75	Urine	PRJNA1102395	SAMN41036354	SRR28760764
19AR0685	ST131	2019	Human	30 to <35	Feces	PRJNA1102395	SAMN41036356	SRR28760762
19AR0752	ST131	2019	Human	45 to <50	Urine	PRJNA1102395	SAMN41036364	SRR28760753
20AR0308	ST131	2020	Human	95 to <100	Urine	PRJNA1097666	SAMN45084036	SRR31540359
20AR0415	ST131	2020	Human	45 to <50	Screen	PRJNA1097666	SAMN45084037	SRR31540358
20AR0429	ST131	2020	Human	85 to <90	Urine	PRJNA1097666	SAMN45084038	SRR31540352
20AR0569	ST131	2020	Human	70 to <75	Feces	PRJNA1097666	SAMN45084039	SRR31540351
20AR0734	ST131	2020	Human	80 to <85	Urine	PRJNA1097666	SAMN45084040	SRR31540350
20AR0735	ST131	2020	Human	70 to <75	Urine	PRJNA1097666	SAMN45084041	SRR31540349
20AR0782	ST131	2020	Human	70 to <75	Urine	PRJNA1097666	SAMN45084042	SRR31540348
20AR1045	ST131	2020	Human	60 to <65	Feces	PRJNA1097666	SAMN45084043	SRR31540347
20AR1061	ST131	2020	Human	65 to <70	Urine	PRJNA1097666	SAMN45084044	SRR31540346
21AR0275	ST131	2021	Human	80 to <85	Blood	PRJNA1097666	SAMN45084045	SRR31540345
21AR0707	ST131	2021	Human	65 to <70	Tissue	PRJNA1097666	SAMN45084046	SRR31540357
22AR0010	ST131	2022	Human	90 to <95	Wound	PRJNA1097666	SAMN45084047	SRR31540356
22AR0042	ST131	2022	Human	35 to <40	Urine	PRJNA1097666	SAMN45084048	SRR31540355
23AR0006	ST131	2023	Human	80 to <85	Feces	PRJNA1097666	SAMN45084049	SRR31540354
23AR0007	ST131	2023	Human	75 to <80	Urine	PRJNA1097666	SAMN45084050	SRR31540353

Appendix Table 4. Illumina quality control metrics for the 33 *Escherichia coli* genomes

	Raw data		Quality control		Taxonomic classification								*de novo* assembly					
Genome ID	Avg. read length (base pair)	Total reads (pairs)	Avg. read length (base pair)2	Total reads (pairs)2	No. reads unclassified (%)	No. reads species #1 (%)	Species #1	No. reads species #2 (%)	Species #2	No. reads species #3 (%)	Species #3	Estimated coverage	No. contigs	No. contigs (min = 1,000)	Largest contig (base pairs)	Chromosome length (base pairs)	GC (%)	N50
18AR0845	149	1,225,228	148	1,212,020	340 (0.03)	594950 (49.09)	*E. coli*	4249 (0.35)	*Ent. cloacae*	1269 (0.1)	*E. albertii*	79	95	90	490,615	5,029,160	50.77	138,708
18AR0858	149	898,997	148	892,155	156 (0.02)	445689 (49.96)	*E. coli*	2964 (0.33)	*Ent. cloacae*	806 (0.09)	*E. albertii*	58	146	127	429,428	5,018,705	50.81	93,538
18AR0998	148	7,122,030	145	6,892,159	4932 (0.07)	3320323 (48.18)	*E. coli*	23419 (0.34)	*Ent. cloacae*	9125 (0.13)	*E. albertii*	458	115	101	435,660	5,007,399	50.78	156,795
18AR1017	149	5,922,271	145	5,738,577	3827 (0.07)	2739510 (47.74)	*E. coli*	19590 (0.34)	*Ent. cloacae*	7470 (0.13)	*E. albertii*	382	96	86	596,001	5,031,176	50.77	183,445
18AR1089	144	1,282,534	142	1,265,196	1176 (0.09)	605806 (47.88)	*E. coli*	1961 (0.15)	*Ent. cloacae*	1774 (0.14)	*Homo sapiens*	80	98	89	364,866	5,013,937	50.76	156,840
18AR1367	148	2,318,372	144	2,234,278	1307 (0.06)	1057814 (47.34)	*E. coli*	7165 (0.32)	*Ent. cloacae*	3237 (0.14)	*E. albertii*	148	106	94	261,299	5,028,794	50.77	156,335
18AR1368	148	2,167,713	144	2,088,357	1418 (0.07)	963487 (46.14)	*E. coli*	9119 (0.44)	*Ent. cloacae*	3049 (0.15)	*E. albertii*	139	112	101	331,814	5,047,023	50.78	134,417
18AR1454	148	3,994,523	146	3,900,580	1807 (0.05)	1845027 (47.3)	*E. coli*	13653 (0.35)	*Ent. cloacae*	4598 (0.12)	*Shigella sp.*	257	95	81	435,572	5,052,408	50.77	160,162
19AR0090	149	2,630,629	146	2,548,939	1548 (0.06)	1245124 (48.85)	*E. coli*	9774 (0.38)	*Ent. cloacae*	3716 (0.15)	*E. albertii*	169	101	91	522,027	5,025,942	50.77	183,445
19AR0427	141	2,771,278	140	2,692,209	1048 (0.04)	1222255 (45.4)	*E. coli*	5353 (0.2)	*Ent. cloacae*	2584 (0.1)	*E. albertii*	170	100	87	393,201	5,025,648	50.76	158,934
19AR0428	139	3,116,457	137	3,019,280	1468 (0.05)	1303265 (43.16)	*E. coli*	6381 (0.21)	*Ent. cloacae*	2795 (0.09)	*E. albertii*	187	93	81	393,201	5,013,631	50.78	156,795
19AR0523	144	2,197,567	143	2,142,463	698 (0.03)	924097 (43.13)	*E. coli*	8130 (0.38)	*Ent. cloacae*	1741 (0.08)	*E. albertii*	137	158	136	282,275	5,186,786	50.75	106,415
19AR0649	146	2,904,352	144	2,837,968	1038 (0.04)	1343104 (47.33)	*E. coli*	9408 (0.33)	*Ent. cloacae*	2753 (0.1)	*E. albertii*	184	95	84	596,343	5,039,079	50.78	158,934
19AR0650	141	3,519,807	139	3,424,356	1737 (0.05)	1456974 (42.55)	*E. coli*	13686 (0.4)	*Ent. cloacae*	2407 (0.07)	*E. albertii*	215	105	92	596,455	5,031,776	50.77	138,652
19AR0667	133	2,244,647	132	2,179,486	1487 (0.07)	882730 (40.5)	*E. coli*	4446 (0.2)	*Ent. cloacae*	2987 (0.14)	*Salm. enterica*	129	101	93	393,201	5,079,645	50.73	135,594
19AR0678	139	1,905,041	138	1,854,738	907 (0.05)	822190 (44.33)	*E. coli*	3867 (0.21)	*Ent. cloacae*	1551 (0.08)	*E. albertii*	114	99	88	596,455	5,033,258	50.77	158,934
19AR0685	138	2,450,090	137	2,383,431	1410 (0.06)	1019458 (42.77)	*E. coli*	7145 (0.3)	*Ent. cloacae*	1896 (0.08)	*E. albertii*	147	99	89	393,202	5,033,357	50.77	181,189
19AR0752	137	3,305,107	135	3,215,026	1885 (0.06)	1315550 (40.92)	*E. coli*	11551 (0.36)	*Ent. cloacae*	2477 (0.08)	*E. albertii*	196	98	88	393,196	5,024,973	50.76	156,795
20AR0308	149	2,934,190	147	2,877,884	1572 (0.05)	1508798 (51.42)	*E. coli*	26802 (0.91)	*Klebs. pneumoniae*	3210 (0.11)	*Shigella sp.*	174	101	90	304,524	5,066,159	50.7	156,840
20AR0415	151	1,257,102	149	1,158,686	476 (0.04)	633125 (50.36)	*E. coli*	9719 (0.77)	*Klebs. pneumoniae*	3149 (0.25)	*Staph. aureus*	75	113	101	257,887	4,907,442	50.77	109,278
20AR0429	151	1,401,872	148	1,182,509	381 (0.03)	679997 (48.51)	*E. coli*	12606 (0.90)	*Staph. aureus*	9167 (0.65)	*Klebs. pneumoniae*	84	133	122	304,523	5,101,075	50.77	95,786
20AR0569	149	1,217,624	145	1,181,038	1012 (0.08)	647987 (53.22)	*E. coli*	9928 (0.82)	*Klebs. pneumoniae*	1381 (0.11)	*E. albertii*	72	173	160	207,614	5,057,120	50.78	69,369

	Raw data		Quality control		Taxonomic classification								*de novo* assembly					
Genome ID	Avg. read length (base pair)	Total reads (pairs)	Avg. read length (base pair)2	Total reads (pairs)2	No. reads unclassified (%)	No. reads species #1 (%)	Species #1	No. reads species #2 (%)	Species #2	No. reads species #3 (%)	Species #3	Estimated coverage	No. contigs	No. contigs (min = 1,000)	Largest contig (base pairs)	Chromosome length (base pairs)	GC (%)	N50
20AR0734	149	1,912,306	146	1,863,053	1693 (0.09)	1029738 (53.85)	*E. coli*	23450 (1.23)	*Klebs. pneumoniae*	1940 (0.10)	*E. albertii*	113	124	113	288,854	5,001,919	50.78	110,297
20AR0735	149	1,624,549	146	1,582,019	1911 (0.12)	838071 (51.59)	*E. coli*	19338 (1.19)	*Klebs. pneumoniae*	11431 (0.70)	*E. marmotae*	96	122	109	236,467	5,102,973	50.76	109,621
20AR0782	148	5,524,013	143	5,347,465	5574 (0.10)	2948287 (53.37)	*E. coli*	7956 (0.14)	*Klebs. pneumoniae*	6309 (0.11)	*E. albertii*	327	118	106	259,946	5,019,794	50.78	135,598
20AR1045	149	3,276,121	144	3,167,405	4285 (0.13)	1707629 (52.12)	*E. coli*	34405 (1.05)	*Klebs. pneumoniae*	4102 (0.13)	*E. albertii*	194	105	91	253,322	5,023,543	50.76	116,479
20AR1061	149	3,497,436	144	3,392,902	3314 (0.09)	1864260 (53.30)	*E. coli*	37224 (1.06)	*Klebs. pneumoniae*	4011 (0.11)	*Shigella sp.*	208	90	82	342,084	5,010,063	50.78	156,623
21AR0275	146	1,879,976	141	1,807,659	6166 (0.33)	938168 (49.90)	*E. coli*	16246 (0.86)	*Klebs. pneumoniae*	4604 (0.24)	*Salm. enterica*	109	116	104	304,523	5,087,184	50.73	138,652
21AR0707	149	3,572,905	143	3,451,166	4731 (0.13)	1818619 (50.90)	*E. coli*	37603 (1.05)	*Klebs. pneumoniae*	5145 (0.14)	*Salm. enterica*	212	107	97	304,523	5,146,465	50.65	138,707
22AR0010	148	1,684,538	144	1,638,600	1875 (0.11)	852148 (50.59)	*E. coli*	21737 (1.29)	*Klebs. pneumoniae*	1890 (0.11)	*Salm. enterica*	99	133	114	304,523	5,110,615	50.77	135,594
22AR0042	142	2,930,447	137	2,811,838	7866 (0.27)	1416623 (48.34)	*E. coli*	21399 (0.73)	*Klebs. pneumoniae*	3832 (0.13)	*E. albertii*	165	97	85	342,091	5,005,323	50.77	134,925
23AR0006	147	2,114,716	139	1,983,903	4040 (0.19)	1051351 (49.72)	*E. coli*	27475 (1.30)	*Klebs. pneumoniae*	3473 (0.16)	*Salm. enterica*	124	113	98	353,024	4,965,594	50.75	116,868
23AR0007	147	1,635,896	139	1,533,412	2791 (0.17)	815713 (49.86)	*E. coli*	11935 (0.73)	*Klebs. pneumoniae*	3873 (0.24)	*Salm. enterica*	95	119	106	295,718	5,121,264	50.72	110,810

Appendix Table 5. Quality control metrics for nanopore sequencing of sample 18AR0845

Category	Type	Value
Raw data	Median read length	2,242
	Median read quality	9.7
	Number of reads	197,452
	Read length N50	6,950
	Total number of bases	768,738,047
	Bio Project ID	PRJNA1102395
	BioSample	SAMN41036272
	Nanopore SRA	SRR31614413
Quality control*	Median read length	2,138
	Median read quality	9.9
	Number of reads	197,323
	Read length N50	7,010
	Total number of bases	747,897,984
	No. reads unclassified (%)	4,366 (2.21)
	No. reads species #1 (%)	143,706 (72.83)
	Species #1	*Escherichia coli*
	No. reads species #2 (%)	4,144 (2.10)
	Species #2	*Klebsiella pneumoniae*
	No. reads species #3 (%)	1,322 (0.67)
	Species #3	*Salmonella enterica*
	Estimated coverage	162x
de novo assembly	No. contigs	7
	Largest contig (base pairs)	4,974,820
	Total length (base pairs)	5,125,043
	GC (%)	50.79
Typing	MLST	ST131
	Serotype	O25b:H4
	*fim*H type	*fim*H30
	No. of prophage elements	7
	AMR genes	
	Chromosome	*pmr*B_E123D, *bla*EC, *mdt*M, *emr*E, *gyr*A_D87N, *gyr*A_S83L, *glp*T_E448K, *pts*I_V25I, *par*C_E84V, *par*C_S80I, *par*E_I529L, *acr*F, *uhp*T_E350Q, *emr*D
	Plasmid 1	*dfr*A17, *aad*A5, *sul*1, *mph*(A), *sul*2, *aph*(3”)-Ib, *aph*(6)-Id, *tet*(A), *bla*CTX-M-174
	Plasmid 2	*bla*OXA-48
	Virulence genes	
	Chromosome	*fde*C, *iss*, *ari*R, *ybt*Q, *ybtP*, *pap*A, *iuc*A, *iuc*B, *iuc*C, *iuc*D, *iut*A, sat, *iha*
	Plasmid 1	*sen*B, *qac*Edelta1

*Removing the first and last 52bp from each read; removing any reads with a Q score <7.

Appendix Table 6. Whole-genome sequences of 22 publicly-available *Escherichia coli* genomes used in this investigation: Metadata

ID	Country	Collection date	Source	Sample	Bio Project ID	BioSample	SRA
DRR387864	Japan	2019	Human	Urine	PRJDB10842	SAMD00499360	DRR387864
DRR389782	Japan	2019	Human	Feces	PRJDB10842	SAMD00501278	DRR389782
DRR389827	Japan	2019	Human	-	PRJDB10842	SAMD00501323	DRR389827
4119STDY6381687	Vietnam	2012/2013	Human	Rectal swab	PRJEB12887	SAMEA4061289	ERR1789811
4119STDY6381688	Vietnam	2012/2013	Human	Rectal swab	PRJEB12887	SAMEA4061292	ERR1789812
4119STDY6380012	Vietnam	2012/2013	Human	Rectal swab	PRJEB12887	SAMEA3980738	ERR1681734
4119STDY6382884	Vietnam	2012/2013	Human	Rectal swab	PRJEB12887	SAMEA4062188	ERR1791000
4119STDY6382885	Vietnam	2012/2013	Human	Rectal swab	PRJEB12887	SAMEA4062197	ERR1791001
4119STDY6382888	Vietnam	2012/2013	Human	Rectal swab	PRJEB12887	SAMEA4062233	ERR1791004
4406STDY6581229	Vietnam	2011/2013	Human	Blood	PRJEB15430	SAMEA44702778	ERR1852604
4406STDY6620929	Vietnam	2011/2013	Human	Blood	PRJEB15430	SAMEA4552134	ERR1937152
ERR1971652	Denmark	2014	Human	Blood	PRJEB20792	SAMEA104060709	ERR1971652

ID	Country	Collection date	Source	Sample	Bio Project ID	BioSample	SRA
ERR2238055	Ireland	2016	Human	-	PRJEB21277	SAMEA104458080	ERR2238055
ERR2060139	Vietnam	2012/2013	Human	Feces	PRJEB21997	SAMEA104188787	ERR2060139
ERR2538197	Cambodia	2016	Human, food	-	PRJEB25898	SAMEA1061957	ERR2538197
ERR2538552	Cambodia	2016	Human, food	-	PRJEB25898	SAMEA1062085	ERR2538552
ERR4221000	Thailand	2018	Human	Feces	PRJEB38313	SAMEA6832590	ERR4221000
ERR5037395	France	2015	Human	-	PRJEB42322	SAMEA7758297	ERR5037395
ERR5037306	France	2018	Human	-	PRJEB42322	SAMEA7758208	ERR5037306
SRR5936518	Singapore	2015	Human	Blood	PRJNA514245	SAMN07510268	SRR5936518
SRR19561770	Australia	2018	Human	Blood	PRJNA797179	SAMN28669217	SRR19561770
SRR19091379	Thailand	2017	Human	Urine	PRJNA814829	SAMN28097299	SRR19091379

Appendix Table 7. Mobile genetic elements and predicted recombination sites relative to the chromosome of sample 18AR0845

Description	Start	Stop	Size (bp)	Number of single-nucleotide variants
ISKpn8	165,126	166,568	1,442	0
ISEc52	420,788	422,037	1,249	0
ISEc52	620,384	621,633	1,249	0
cn_44780_IS4	779,415	824,195	44,780	16
Recombination	813,139	813,305	166	0
Recombination	833,387	833,740	353	0
IS682	834,798	837,329	2,531	0
IS30	846,475	847,695	1,220	0
IS629	866,393	867,702	1,309	0
Recombination	872,234	872,458	224	0
MITEEc1	923,403	923,524	121	0
MITEEc1	1,754,983	1,755,105	122	0
cn_35467_IS682	1,880,736	1,916,203	35,467	0
Recombination	1,904,322	1,904,438	116	0
cn_7294_IS682	1,913,671	1,920,965	7,294	0
IS682	1,913,672	1,916,203	2,531	0
Prophage 1	2,022,670	2,053,189	30,519	4
Prophage 2	2,049,142	2,077,029	27,887	3
ISEc1	2,471,491	2,472,781	1,290	0
ISKpn8	2,502,128	2,503,570	1,442	0
Prophage 3	2,631,468	2,686,293	54,825	3
Prophage 4	2,735,190	2,778,911	43,721	2
ISEc52	2,823,606	2,824,855	1,249	0
Prophage 5	2,829,772	2,874,626	44,854	7
Prophage 6	3,075,245	3,138,785	63,540	3
MITEEc1	3,275,147	3,275,269	122	0
Recombination	3,729,121	3,729,172	51	3
ISEc53	4,181,983	4,183,867	1,884	0
cn_42182_ISEc52	4,190,086	4,232,268	42,182	5
Recombination	4,210,238	4,210,856	618	0
Recombination	4,222,090	4,226,852	4,762	0
Recombination	4,231,498	4,231,629	131	0
ISSfl10	4,232,269	4,233,533	1,264	0
cn_2977_IS682	4,244,016	4,246,993	2,977	0
Prophage 7	4,705,929	4,740,049	34,120	1

Appendix Table 8. *Escherichia coli* BEAST analysis results summary*

		BEAST2 run outputs					
Clock Rate model	Population model	Chain length for each triplicate	tMRCA (95% HPD)	Mean clock rate (95% HPD)	Mean tree likelihood	Median clock rate	SNP/year/site†
Relaxed log-normal	Bayesian skyline	100 million	2006 (1999 to 2010)	6.57E-03 (4.41E-03 to 8.91E-03)	−2,228.65	6.46E-03	4.38E-07
Relaxed log-normal	Bayesian skyline	100 million	2006 (1993 to 2009)	6.83E-03 (4.57E-03 to 9.25E-03)	−2,232.14	6.75E-03	4.57E-07
Relaxed log-normal	Bayesian skyline	100 million	2004 (1994 to 2009)	6.79E-03 (4.41E-03 to 9.31E-03)	−2,232.45	6.69E-03	4.53E-07

*10% Burnin and 1,000 replicates. HPD, highest probability density; SNP, single-nucleotide variant; tMRCA, time to most recent common ancestor.
†Relative to genome size.

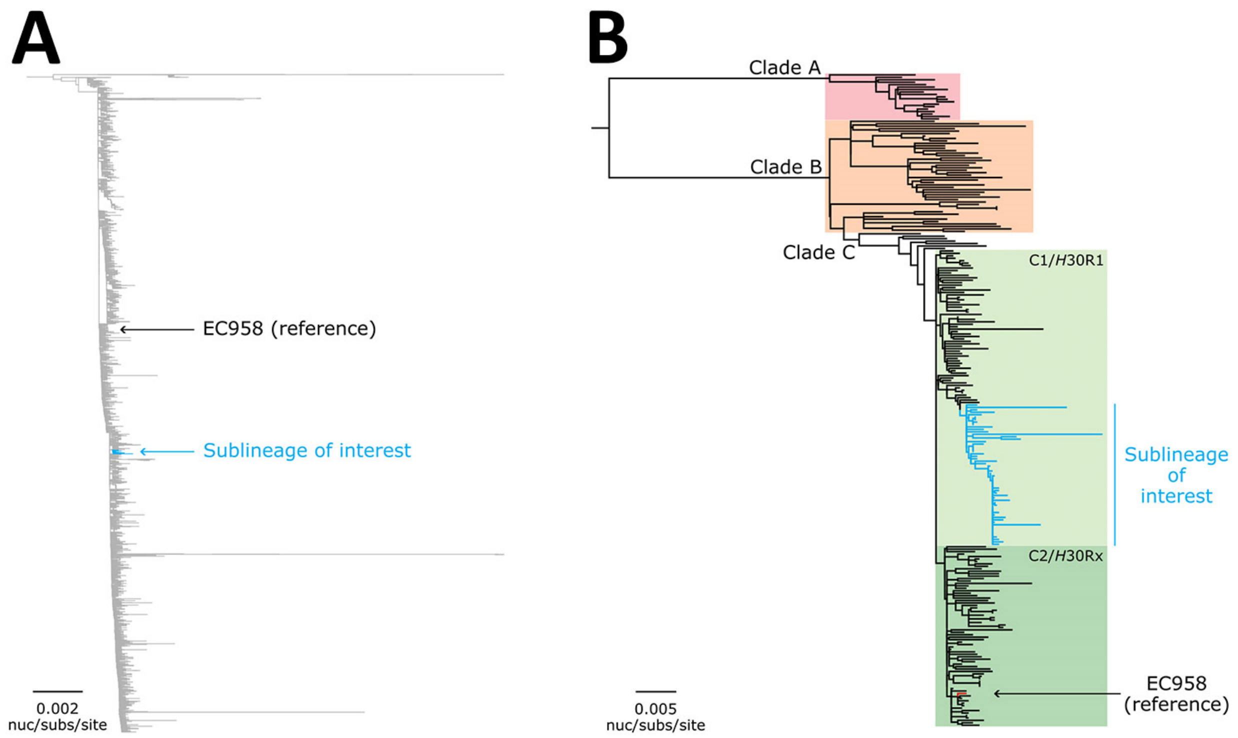

Appendix Figure 1. Maximum-likelihood phylogeny for *Escherichia coli* sequence type (ST)131 genomes. (a) The phylogeny was inferred from 8,950 core-genome single-nucleotide variants (SNVs) from 12,185 genomes. SNVs were derived from a core-genome alignment of 550,364 bp (b) The phylogeny was inferred from 3,741 non-recombinant core-genome SNVs from 264 genomes. SNVs were derived from a core-genome alignment of 3,392,993 bp. In both analyses, SNVs were called against the chromosome of EC958 (GenBank: HG941718).

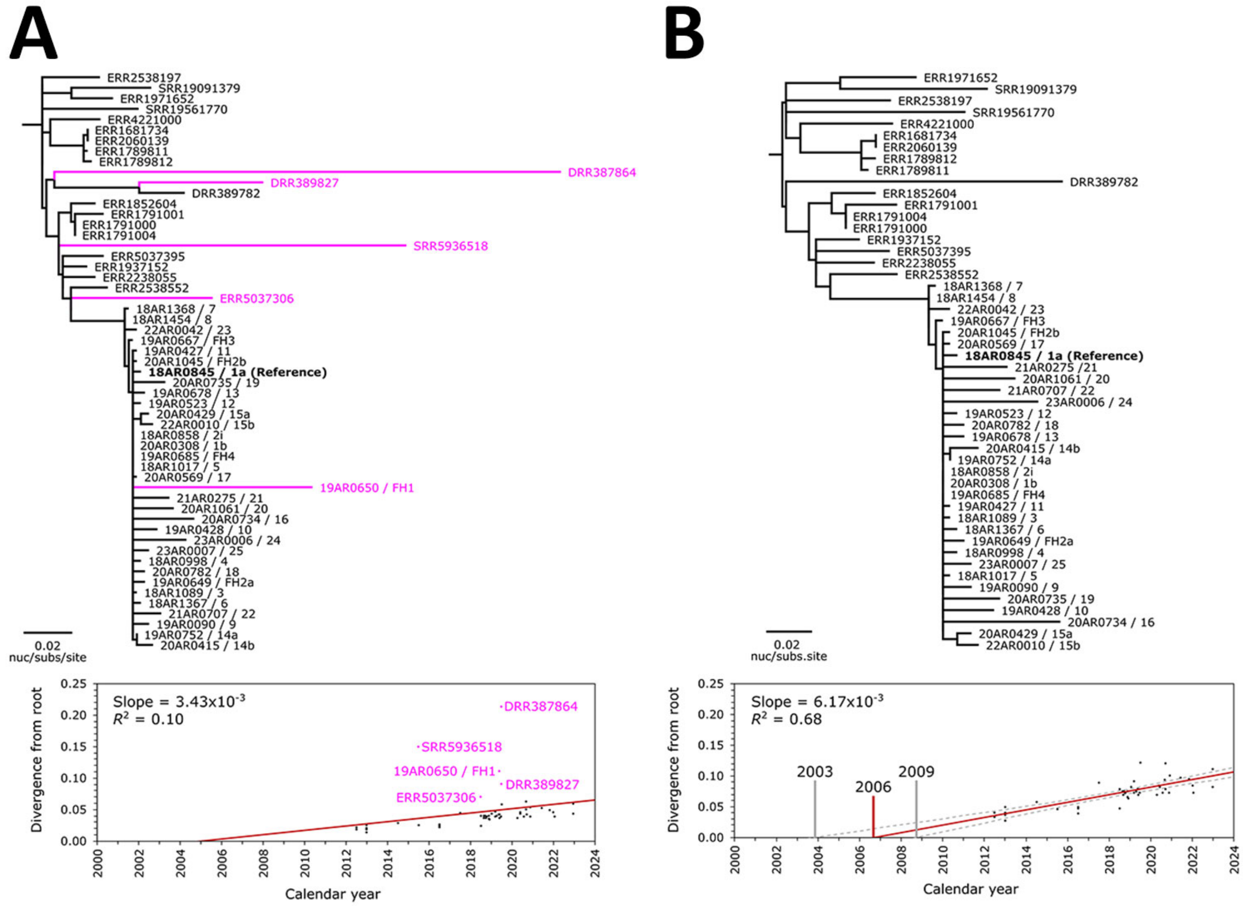

Appendix Figure 2. Maximum-likelihood phylogeny for OXA-48-producing *Escherichia coli* sequence type (ST)131 genomes obtained from cases and food handlers in relation to a cluster investigation, Hutt Valley, New Zealand, 2018–2022, compared with publicly available genomes. (**a**) The phylogeny was inferred from 602 non-recombinant orthologous biallelic core-genome single-nucleotide variants (SNVs) from 55 genomes. SNVs were derived from a core-genome alignment of ~4,599,200 bp (**b**) The phylogeny was inferred from 323 non-recombinant orthologous biallelic core-genome SNVs from 50 genomes. SNVs were derived from a core-genome alignment of ~4,767,900 bp. In both analyses, SNVs were called against the chromosome of 18AR0845 (GenBank: CP175691). Both phylogenetic trees are rooted according to the ERR1822501 outgroup, which has been omitted for visualization.
